# Structure and Dielectric Properties of Poly(vinylidenefluoride-co-trifluoroethylene) Copolymer Thin Films Using Atmospheric Pressure Plasma Deposition for Piezoelectric Nanogenerator

**DOI:** 10.3390/nano13101698

**Published:** 2023-05-22

**Authors:** Eunyoung Jung, Choon-Sang Park, Taeeun Hong, Heung-Sik Tae

**Affiliations:** 1 The Institute of Electronic Technology, College of IT Engineering, Kyungpook National University, Daegu 41566, Republic of Korea; eyjung@knu.ac.kr; 2 Department of Electrical Engineering, Milligan University, Johnson City, TN 37682, USA; cpark@milligan.edu; 3 Division of High-Technology Materials Research, Korea Basic Science Institute, Busan 46742, Republic of Korea; tehong@kbsi.re.kr; 4 School of Electronic and Electrical Engineering, College of IT Engineering, Kyungpook National University, Daegu 41566, Republic of Korea

**Keywords:** P[VDF–TrFE], atmospheric pressure plasmas, plasma deposition, dielectric constant

## Abstract

This study investigates the structural phase and dielectric properties of poly(vinylidenefluoride-co-trifluoroethylene) (P[VDF–TrFE]) thin films grown via atmospheric pressure (AP) plasma deposition using a mixed polymer solution comprising P[VDF–TrFE] polymer nano powder and dimethylformamide (DMF) liquid solvent. The length of the glass guide tube of the AP plasma deposition system is an important parameter in producing intense cloud-like plasma from the vaporization of DMF liquid solvent containing polymer nano powder. This intense cloud-like plasma for polymer deposition is observed in a glass guide tube of length 80 mm greater than the conventional case, thus uniformly depositing the P[VDF–TrFE] thin film with a thickness of 3 μm. The P[VDF–TrFE] thin films with excellent β-phase structural properties were coated under the optimum conditions at room temperature for 1 h. However, the P[VDF–TrFE] thin film had a very high DMF solvent component. The post-heating treatment was then performed on a hotplate in air for 3 h at post-heating temperatures of 140 °C, 160 °C, and 180 °C to remove DMF solvent and obtain pure piezoelectric P[VDF–TrFE] thin films. The optimal conditions for removing the DMF solvent while maintaining the β phases were also examined. The post-heated P[VDF–TrFE] thin films at 160 °C had a smooth surface with nanoparticles and crystalline peaks of β phases, as confirmed by the Fourier transform infrared spectroscopy and XRD analysis. The dielectric constant of the post-heated P[VDF–TrFE] thin film was measured to be 30 using an impedance analyzer at 10 kHz and is expected to be applied to electronic devices such as low-frequency piezoelectric nanogenerators.

## 1. Introduction

Dielectric, piezoelectric, and ferroelectric polymer materials have recently received immense attention for their application in flexible electronics, such as sensors, actuators, capacitors, and energy storage devices [1,2]. Due to the importance of dielectric materials, new, very precise quartz methods of dielectric measurement have also been developed, which also take temperature compensation into account [3,4]. As a result, these polymers have been applied in industrial electronic devices instead of piezoelectric ceramics [2]. Thus, polyvinylidene fluoride (PVDF) and its copolymers have attracted attention in the field of piezoelectric nanogenerator devices owing to their mechanical flexibility, lightweight, piezoelectricity, dielectric property, thermal stability, high chemical resistance, and good biocompatibility [5,6,7]. This PVDF and its copolymers are semicrystalline polymers that have piezoelectric property due to the carbon and fluorine chains [8,9]. Because the TrFE unit in poly(vinylidenefluoride-co-trifluoroethylene) (P[VDF–TrFE]) allows the copolymer to have a ferroelectric β phase at room temperature, it shows strong ferroelectric properties [10,11]. However, the dielectric constant and piezoelectricity of these piezoelectric polymers are relatively low compared to ceramic substances, such as barium titanate and lead zirconate titanate [12,13,14]. Thus, the development of piezoelectric polymers with a high dielectric constant is necessary to industrialize the application of piezoelectric polymers. Many research efforts have been directed toward developing piezoelectric polymer materials, such as copolymers and polymer nanocomposites containing piezoelectric ceramic nanoparticles [15,16,17,18]. In previous studies, only material properties were examined [15,16,17,18]. These piezoelectric polymers can be mostly fabricated by using a conventional method, such as inkjet printing, screen printing, electrospinning, and spin-coating [19,20]. However, there have been few studies on atmospheric pressure (AP) plasma other than the conventional method, such as electrospinning and spin casting [19,20].

In this study, we investigated the structural phase and dielectric properties of P[VDF–TrFE] thin films grown via AP plasma deposition using a mixed polymer solution comprising P[VDF–TrFE] polymer nano powder and dimethylformamide (DMF) liquid solvent. The lengths of the glass guide tube of the AP plasma deposition system were examined to produce intense cloud-like plasma from the vaporization of DMF liquid solvent containing polymer powder. The plasma properties were investigated using a digital camera and an intensified charge-coupled device (ICCD). Furthermore, to remove DMF solvent and obtain pure piezoelectric P[VDF–TrFE] thin films, we investigated the β-phase structural properties of P[VDF–TrFE] thin films grown via the newly proposed AP plasma deposition with a mixed polymer solution comprising P[VDF–TrFE] polymer nano powder and DMF liquid solvent. Particularly, the structural phase and dielectric properties of P[VDF–TrFE] thin film were examined under post-heating temperatures of 140 °C, 160 °C, and 180 °C using field emission scanning electron spectroscopy (FE-SEM), X-ray diffractometer (XRD), Fourier transforms infrared spectroscopy (FT-IR), stylus profiler, and impedance analyzer.

## 2. Materials and Methods

### 2.1. Experimental Setup

P[VDF–TrFE] nano powder (F50, Piezotech, Pierre-Bénite, France) blended with a condition of VDF/TrFE ratios of VDF 50%mol and TrFE 50%mol was used. The used P[VDF–TrFE] nano powder has a melting temperature of about 200 °C and very low vapor pressure [8,9,10,11]. Consequently, the concentration of this precursor in the plasma chamber cannot be high and, furthermore, cannot be well controlled by flow of carrier gas. Hence, unlike the liquid monomer, it is very difficult to increase the concentration of this precursor in the plasma chamber by just manipulating the flow of carrier gas. In this study, a mixed polymer solution with a low P[VDF–TrFE] nano powder concentration (below 5%) was used, in which P(VDF–TrFE) nano powder was dissolved in a DMF solvent in order to sufficiently vaporize it by Ar gas flow. If the mixed P[VDF–TrFE] polymer solution has a high concentration over 5%, the Ar gas bubbling for vaporizing the P[VDF–TrFE] polymer is very difficult due to its high viscosity. Furthermore, it was observed that increasing Ar gas flow had little effect on vaporizing the P(VDF–TrFE) polymer under the high concentration condition (>5%). Providing the high concentration of the precursor in the plasma chamber requires further study. Therefore, the P[VDF–TrFE] nano powder concentration of 5% was an optimal condition for preparing a mixed P[VDF–TrFE] polymer solution. To prepare a 5% P[VDF–TrFE] polymer solution, 1.95 g of P[VDF–TrFE] polymer nano powder was diluted in 40 mL of DMF liquid solvent. For P[VDF–TrFE] polymer film deposition, the vaporized polymer was formed by an Ar gas flow using a mixed polymer solution. The vaporized polymer was injected into the plasma reactor as a precursor for polymer film deposition.

The polymer solutions were uniformly mixed on a hotplate at 40 °C for 24 h using a magnetic stirring bar at an angular speed of 500 rpm to ensure complete dissolution.

**Figure 1:**
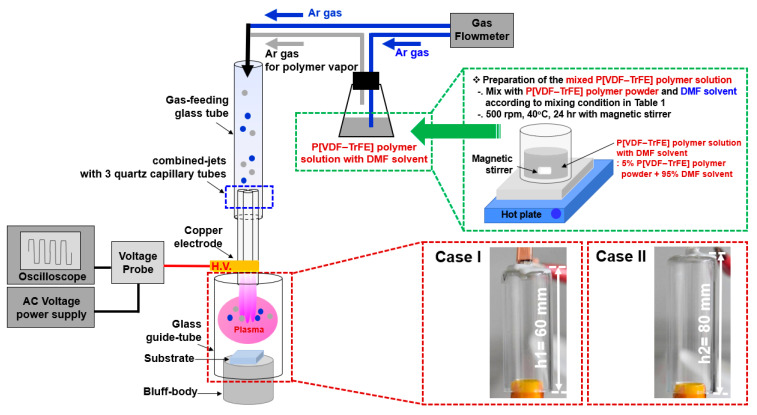
Experimental setup of atmospheric pressure (AP) plasma deposition system employed in this study and the photo images of glass guide tubes with different heights (h1 = 60 mm (case I)) and h2 = 80 mm (case II)), as shown in Table 1.

Figure 1 depicts the experimental setup of the AP plasma deposition system employed in this study using a mixed polymer solution comprising P[VDF–TrFE] polymer nano powder and DMF solvent for P[VDF–TrFE] thin film deposition. In our previous publication, we described in detail the experimental setup of the AP plasma deposition system [21,22,23,24]. This AP plasma deposition technique is a method capable of synthesizing nanoparticles (NPs) or depositing films at room temperature [21,22,23,24]. The AP plasma deposition system comprises a gas feeding tube, a glass guide tube for producing plasma discharge, a bluff body, and combined jets with three quartz capillary tubes. The quartz capillary jets were assembled in a triangular shape and attached with copper tape as a high-voltage electrode. The bluff body was made of polytetrafluoroethylene material, and the substrate was placed on the bluff body inside the glass guide tube.

The mixed polymer solution with DMF solvent was vaporized via argon (Ar) gas flow for the AP plasma deposition. The plasma discharge and vaporized mixed P[VDF–TrFE]/DMF polymer solution required a flow rate of Ar gas of 2500 standard cubic centimeters per minute (sccm) and 450 sccm, respectively. A sinusoidal voltage was applied with a peak value of 12.5 kV at a frequency of 26 kHz to produce a plasma discharge for depositing the P[VDF–TrFE] thin films. Furthermore, glass and ITO-coated glass were used as substrates in the AP plasma deposition using a mixed polymer solution comprising P[VDF–TrFE] polymer nano powder and DMF solvent.

In order to remove DMF solvent and obtain pure piezoelectric P[VDF–TrFE] thin films, the deposited P[VDF–TrFE] thin film was heated for 3 h on a hot plate in air at post-heating temperatures of 140 °C, 160 °C, and 180 °C. Table 1 lists the detailed experimental conditions for depositing the P[VDF–TrFE] thin film.

### 2.2. Intensified Charge-Coupled Device

The horizontal and vertical spatial distributions of the produced plasma were evaluated using an ICCD camera (PIMAX 2, Princeton Instruments, Trenton, NJ, USA) with an exposure time of 100 ms.

### 2.3. Optical Emission Spectroscopy

To investigate the characteristics of excited radical species such as reactive nitrogen species (RNS) and excited Ar radicals in the formed plasma discharge, optical emission spectroscopy (OES) was obtained using an optical spectrometer (Ocean optics, USB-2000+, Orlando, FL, USA), assembled with a 1 mm diameter optical fiber and a collimating lens. The spectral resolution was 0.06 nm with a wavelength range of 200–900 nm.

### 2.4. Field Emission Scanning Electron Microscopy

The surface morphology of P[VDF–TrFE] thin film was evaluated on the surface structure using an FE-SEM (Hitachi SU8220, Hitachi High-Technologies, Tokyo, Japan) at a voltage and current of 3 kV and 10 μA, respectively. Before loading into the chamber, the samples were made conductive for FE-SEM by coating them with platinum.

### 2.5. X-ray Diffraction

The crystalline structure of the deposited P[VDF–TrFE] thin films was evaluated using X-ray diffraction (XRD; D8 Discover Bruker, Karlsruhe, Germany) at the Korea Basic Science Institute (KBSI; Daegu, Republic of Korea) with 2θ angle in the range of 10°–50° at 0.08 intervals, and CuKa radiation (λ = 1.54 Å) was employed as the X-ray beam source.

### 2.6. Fourier Transformation Infrared Spectroscopy

The main functional groups and crystalline phase of P[VDF–TrFE] thin films were characterized using Fourier transformation infrared spectroscopy (FT-IR; Vertex 70, Bruker, Germany) at the KBSI (Daegu, Republic of Korea). The FT-IR spectra were acquired using attenuated total reflection conditions at a wavenumber resolution interval of 0.6 cm^−1^ in the region from 650 to 4000 cm^–1^.

### 2.7. Stylus Profiler

The film thickness of P[VDF–TrFE] thin film was measured using a stylus profiler (KLA Tencor, P-7, KLA Tencor Corp., Milpitas, CA, USA) at the KBSI (Busan, Republic of Korea), which can directly measure the thickness of thin films using a diamond stylus moving vertically and laterally in contact with the sample.

### 2.8. Impedance Analyzer

To measure the capacitance and dielectric constant, a unit capacitor structure with metal–insulator–metal capacitor films was used as a sandwich type for electrical measurement. The capacitance of P[VDF–TrFE] thin films was measured at room temperature in air using an impedance analyzer (4194A, Agilent, Santa Clara, CA, USA) at a frequency range of 100 Hz to 100 kHz. The dielectric constant was then calculated from the measured capacitance values using the following equation [25]:

C = ε_o_ε_r_A/d,

where C is the measured capacitance, ε_r_ is the dielectric constant, A is the area of the metal electrode, d is the film thickness, and ε_o_ is the permittivity of vacuum. It is necessary to measure the thickness of the thin film to calculate the dielectric constant. The thicknesses of P[VDF–TrFE] thin films were obtained using a stylus profiler.

## 3. Results

In our previous research, a short glass guide tube with a length of 60 mm was commonly used to create strong cloud-like plasma for polymer deposition because a low-molecular-weight monomer solution was used to form the vaporized gas [21,22]. However, in this experiment, plasma must be formed through vaporized polymer using a mixed polymer solution comprising P[VDF–TrFE] polymer nano powder and DMF liquid solvent. The length of the glass guide tube was optimized based on the intensity of the cloud-like plasma produced for different lengths of the glass guide tube in P[VDF–TrFE] thin film deposition.

**Figure 2:**
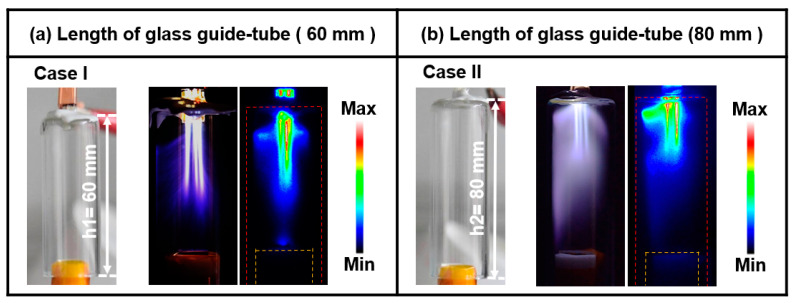
Photographs and intensified charge-coupled device images of plasma obtained by AP plasma deposition from the vaporized polymer precursor using a mixed polymer solution comprising P[VDF–TrFE] polymer nano powder and DMF liquid solvent for two different lengths of glass guide tubes (cases I and II).

Figure 2a,b present photographs and ICCD images of the plasma discharge produced through vaporization of the mixed P[VDF–TrFE] polymer nano powder with DMF solvent in the AP plasma deposition system for two different guide tube lengths (cases I and II). 

In this study, namely, the cases with guide tube lengths of 60 (case I) and 80 mm (case II), were examined. The bluff body was placed at a height of 15 mm inside the guide tube with Ar gas conditions (Ar 2500 sccm and polymer vaporized gas 450 sccm). For case I, the plasma was produced in the form of plasma plumes only, and cloud-like glow plasma was not formed, as shown in Figure 2a. In case II, the plasma discharge was produced by plasma plumes and highly intense cloud-like glow plasma in the guide tube with a mixed P[VDF–TrFE] polymer solution, as shown in Figure 2b. The ICCD image in Figure 2b confirms that the longer guide tube can provide the sufficient plasma discharge required to form the polymer radical species for plasma polymerization when a polymer material with a high molecular weight is used as a precursor, compared to a monomer with a low molecular weight. The experimental results established that the guide tube length of 80 mm (case II) was an optimal length condition for producing an intense cloud-like plasma for P[VDF–TrFE] thin film synthesis.

**Figure 3:**
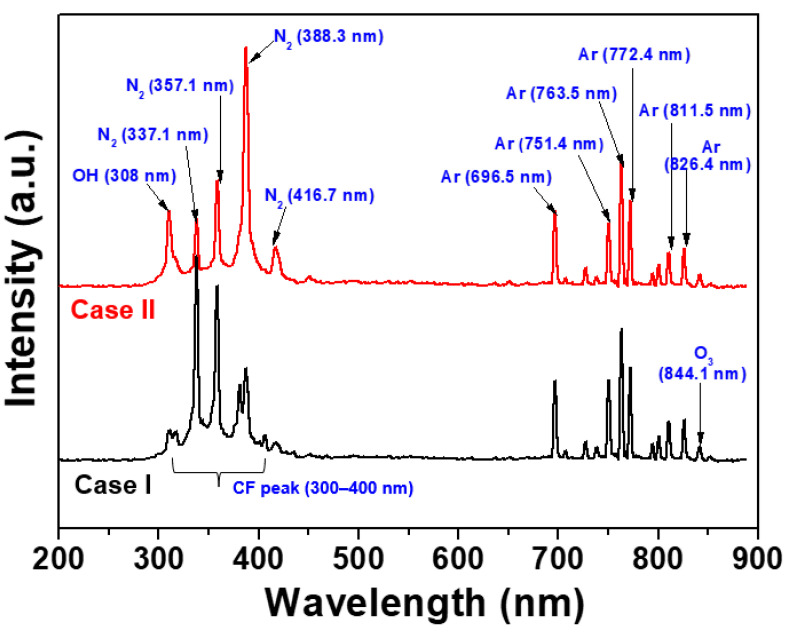
Optical emission spectra measured in the plasma obtained by AP plasma deposition from the vaporized polymer precursor using a mixed polymer solution comprising P[VDF–TrFE] polymer nano powder and DMF liquid solvent for two different lengths of glass guide tubes (cases I and II).

Figure 3 shows the optical emission spectra from 200 to 900 nm, measured in the plasma obtained by AP plasma deposition from the vaporized polymer precursor using a mixed polymer solution comprising P[VDF–TrFE] polymer nano powder and DMF liquid solvent for two different lengths of glass guide tubes (cases I and II). For all OES results, the various nitrogen (N_2_; 337.1, 357.1, 388.3, and 416.7 nm) peaks, oxygen (OH; 308 nm, and O_3_; 844.1 nm) peaks, Ar peaks (Ar; 696.5, 751.4, 763.5, 772.4, 811.5, and 826.4 nm), and broad CF peak (300–400 nm) were observed in the produced plasma [21,23,24,26]. Even if the same amount of the vaporized polymer precursor was injected in the plasma chamber, for case II, the Ar peaks were not changed, and the nitrogen peaks of 388.3, and 416.7 nm predominantly increased due to the collision reaction between Ar gas and vaporized polymer precursor, when compared to case I, as shown in Figure 3. It also indicates that the increase of the radical species contributes to the fragmentation and recombination in the longer guide tube (case II) for P[VDF–TrFE] thin film deposition.

Under the optimal conditions, the P[VDF–TrFE] thin films with excellent β-phase structural properties were successfully coated at room temperature for 1 h in the proposed AP plasma deposition system using the mixed polymer solution. Nevertheless, the P[VDF–TrFE] thin film had a very high DMF solvent component. Thus, to remove the DMF solvent and obtain a pure piezoelectric P[VDF–TrFE] thin film, the post-heating treatment was performed on a hotplate in air for 3 h with three different heating temperatures of 140 °C, 160 °C, and 180 °C.

**Figure 4:**
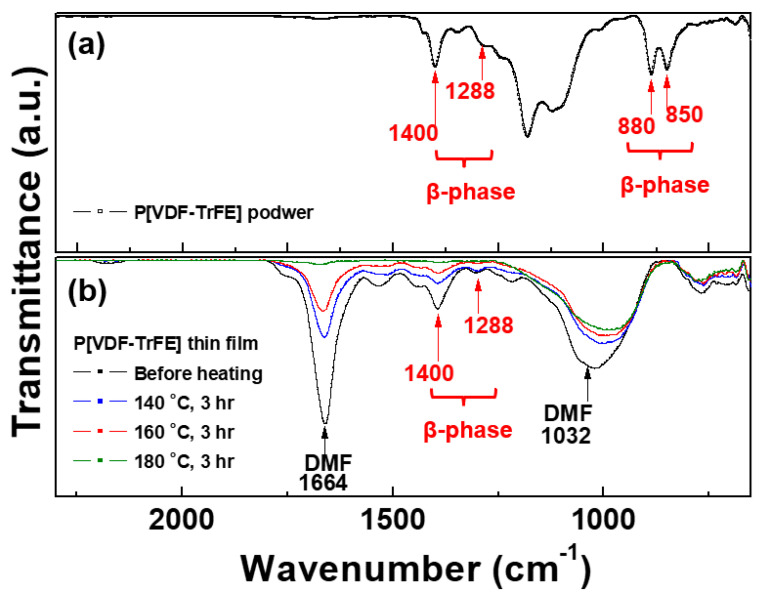
Fourier transforms infrared spectroscopy spectra for (**a**) P[VDF–TrFE] solid nano powder and (**b**) the P[VDF–TrFE] thin films prepared via AP plasma deposition from the vaporized polymer precursor using a mixed polymer solution comprising P[VDF–TrFE] polymer nano powder and DMF liquid solvent at an optimum condition before and after post-heating for 3 h with different post-heating temperature conditions.

As shown in Figure 4a and Table 2, P[VDF–TrFE] solid nano powder has crystalline peaks of β phases, representing the peaks at 1288 and 1400 cm^–1^ for β-phase. The bands at 1400 cm^–1^ are related to the CH_2_ wagging vibration within the polymer chain structure. Moreover, the bands at 850 and 880 cm^–1^ are assigned to the CF_2_ symmetric stretching and CF_2_ in-plane rocking deformations, respectively [11,27,28,29,30].

Figure 4b and Table 2 show crystalline peaks of β phases in all P[VDF–TrFE] thin films, indicating the peaks at 1288 and 1400 cm^–1^. Furthermore, the characteristic peaks caused by DMF solvent primarily had two peaks at 1032 and 1664 cm^–1^, identified as –C–N and –C=O bonds, respectively [31]. As shown in Figure 4b, two peaks caused by the DMF component are observed to be considerably decreased after the post-heating process with increasing heating temperatures of 140 °C, 160 °C, and 180 °C. After post-heating at 140 °C and 160 °C, the peak intensity of β phases decreased slightly; however, these peaks were well maintained. The crystalline peaks of β phases were confirmed to have almost declined due to the changes in the polymer structure caused by high temperature during post-heating at 180 °C for 3 h. Thus, the optimal post-heating condition for removing only DMF components while maintaining the β phases was determined to be 160 °C for 3 h. The retained β phases will increase the dielectric constant of the post-heated P[VDF–TrFE] thin film.

**Figure 5:**
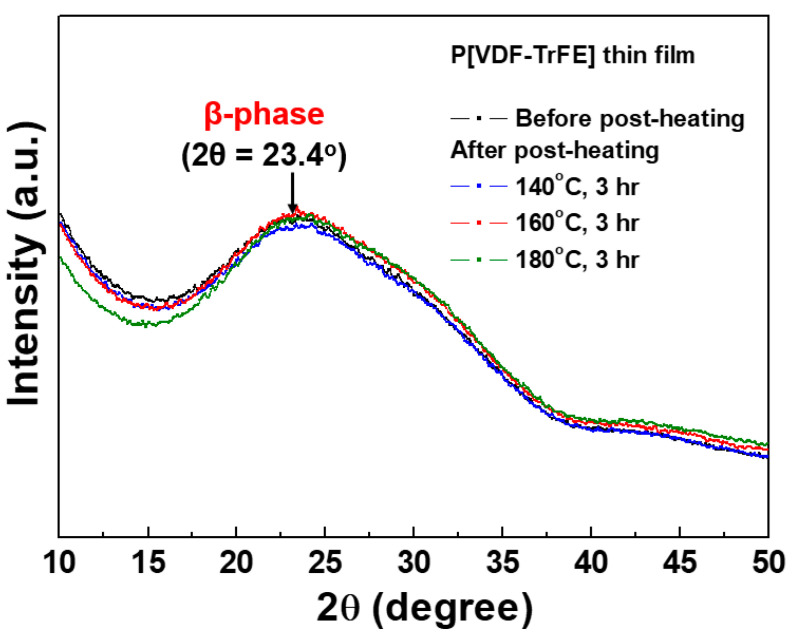
X-ray diffraction patterns for P[VDF–TrFE] thin films coated via AP plasma deposition from the vaporized polymer precursor using a mixed polymer solution comprising P[VDF–TrFE] polymer nano powder and DMF liquid solvent at an optimum condition after post-heating for 3 h with different post-heating temperatures (140 °C, 160 °C, and 180 °C).

Figure 5 shows X-ray diffraction patterns for P[VDF–TrFE] thin films deposited via AP plasma deposition from the vaporized polymer precursor using a mixed polymer solution comprising P[VDF–TrFE] polymer nano powder and DMF liquid solvent before and after post-heating for 3 h at three different post-heating temperatures (140 °C, 160 °C, and 180 °C). In Figure 4, all P[VDF–TrFE] thin films show a wide diffraction peak at 2θ = 23.4°, which is a specific diffraction peak of the β-phase for the (110) and (200) planes [32]. Furthermore, all P[VDF–TrFE] thin films had an amorphous structure due to the long-chain molecules with a higher molecular weight [33]. Thus, the crystallinity in P[VDF–TrFE] thin films weakened, lowering the diffraction peak [33]. After post-heating with increasing heating temperatures, no changes in the diffraction peaks were observed for all P[VDF–TrFE] thin films. Thus, it can be inferred that the post-heating treatment has no considerable effect on the crystal structure of P[VDF–TrFE] thin films.

**Figure 6:**
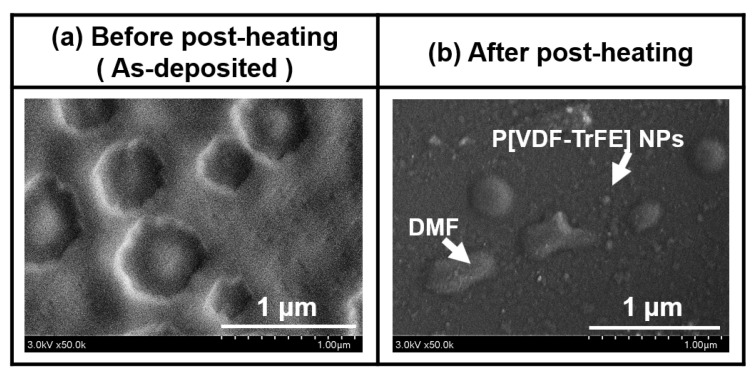
Field emission scanning electron spectroscopy images of P[VDF–TrFE] thin films prepared via AP plasma deposition from the vaporized polymer precursor using a mixed polymer solution comprising P[VDF–TrFE] polymer nano powder and DMF liquid solvent at an optimal condition (**a**) before and (**b**) after post-heating at 160 °C for 3 h.

Figure 6a,b represent the FE-SEM results of P[VDF–TrFE] thin films prepared via AP plasma deposition from the vaporized polymer precursor using a mixed polymer solution comprising P[VDF–TrFE] polymer nano powder and DMF liquid solvent at an optimum condition before and after post-heating at 160 °C for 3 h. The film thickness of post-heated P[VDF–TrFE] thin film was confirmed to be about 3 µm using a stylus profiler.

As shown in Figure 6a, the deposited P[VDF–TrFE] thin film was likely to be formed as a coating layer by the DMF solvent. Before post-heating, the sample was predominantly coated with the DMF layer, and then P[VDF–TrFE] nanoparticles (NPs) were not observed.

After post-heating at 160 °C for 3 h, the deposited film had a smooth surface with P[VDF–TrFE] NPs, as shown in Figure 6b. Moreover, the FE-SEM measurement confirmed a small amount of bubble particles due to DMF solvent attached to the P[VDF–TrFE] thin film.

In general, in the case of increasing a film thickness, piezoelectric polymer film can improve an electrical property, such as dielectric strength and energy density, especially in a piezoelectric nanogenerator [34], whereas, for the optical properties of PVDF-TrFE film, the optical transmission of PVDF-TrFE film is reported to be reduced due to a loss of light, thus resulting in increasing the refractive index with an increase in the film thickness [35]. It will be necessary to carry out further study on effects of the film thickness change through both an increase in the concentration of the precursor and the deposition time on the electrical property for an application of a piezoelectric nanogenerator.

**Figure 7:**
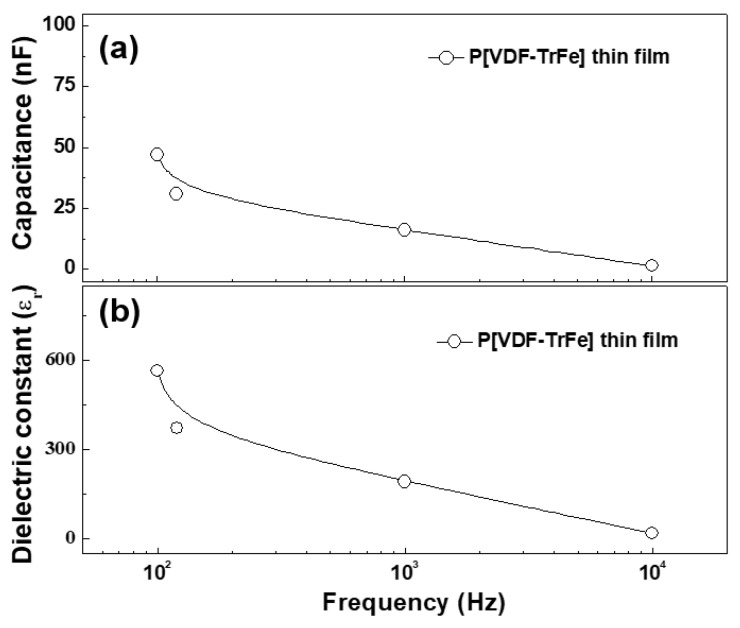
Frequency-dependent variations of the obtained (**a**) capacitance and (**b**) dielectric constant values for P[VDF–TrFE] thin films prepared via AP plasma deposition from the vaporized polymer precursor using a mixed polymer solution comprising P[VDF–TrFE] polymer nano powder and DMF liquid solvent at an optimal condition after post-heating at 160 °C for 3 h.

To investigate the suitability of the piezoelectric polymer thin film for use in a piezoelectric nanogenerator device at a condition of room temperature and low frequency (a few hundred kHz), the capacitance and dielectric constant were measured using an impedance analyzer. If the dielectric properties are required at high temperature and high frequency (over MHz), the detailed dielectric property will be discussed in a future publication with various conditions of high temperature and high frequency. The capacitance and dielectric constant of the post-heated (160 °C) P[VDF–TrFE] thin film were measured at a frequency ranging from 100 Hz to 10 kHz using an impedance analyzer. Figure 7a,b represent the capacitance and dielectric constant values measured in frequency ranging from 100 Hz to 10 kHz for P[VDF–TrFE] thin films on ITO glass substrate deposited via AP plasma deposition after post-heating at 160 °C for 3 h.

In Figure 7a,b, the capacitance and dielectric constant values were observed to be decreased with increasing frequency due to dipole dispersion in polymer structures [36,37]. Generally, at low frequencies (below 1 kHz), the dielectric constant for PVDF and its copolymer usually tends to have a high value due to the polarization orientation caused by the dipole moment inside polymer structures [36,37,38,39,40]. Meanwhile, the dielectric constant decreases with increasing the frequencies (over 1 MHz) due to the dipole dispersion by an oscillated dipoles at a faster speed [36,37,41]. If the dielectric properties are required at high temperature and high frequency (over MHz), the detailed dielectric property will be also discussed in a future publication with various conditions of high temperature and high frequency. Thus, the dielectric constant value of the post-heated P[VDF–TrFE] thin film at 160 °C for 3 h was 30 at 10 kHz and room temperature condition.

The above results suggest that the post-heated P[VDF–TrFE] thin film can be used in piezoelectric nanogenerator devices due to its high capacitance and dielectric constant value. Table 3 shows the dielectric constant values for the post-heated P[VDF–TrFE] thin film at 160 °C for 3 h, measured in frequencies ranging from 100 Hz to 10 kHz. The tangent losses measured in the low-frequency region showed low values similar to those in the literature and the obtained values mentioned in Table 3 [25,36,37,39].

## 4. Conclusions

The present research contributes to the development of material properties of piezoelectric polymer materials to improve the device performance for energy storage applications such as capacitors and nanogenerators. For this purpose, P[VDF–TrFE] thin films were fabricated via a simple AP plasma deposition using a mixed polymer solution comprising P[VDF–TrFE] polymer nano powder and DMF liquid solvent. The length of the guide tube was investigated as the key parameter for producing intense cloud-like plasma discharge in the AP plasma deposition using a mixed polymer solution comprising P[VDF–TrFE] polymer nano powder and DMF liquid solvent. The highly intense cloud-like plasma was formed at an optimum condition of 80 mm (case II), which was confirmed using ICCD. P[VDF–TrFE] thin films with excellent β-phase structural properties were coated using a new AP plasma deposition process under the optimum conditions for 1 h. However, the formed P[VDF–TrFE] thin film had a considerably high DMF solvent component. Furthermore, to remove only the DMF solvent without disturbing the pure piezoelectric P[VDF–TrFE] thin film structure, the post-heating treatment was performed on a hotplate in air for 3 h with three different heating temperatures at 140 °C, 160 °C, and 180 °C. After post-heating at 160 °C for 3 h, DMF peaks were considerably reduced, whereas the β-phase peaks of 1288 and 1400 cm^−1^ were observed to be well maintained. These β-phase peaks enhanced the dielectric constant of the P[VDF–TrFE] thin film. As a result, the post-heated (160 °C) P[VDF–TrFE] thin films had a smooth surface with nanoparticles and crystalline peaks of β phases, which was confirmed using the FT-IR and XRD analyses. The dielectric constant of the post-heated P[VDF–TrFE] thin film was measured to be 30 at 10 kHz using an impedance analyzer. Based on the experimental results, the post-heated P[VDF–TrFE] thin film coated via a simple AP plasma deposition using a mixed polymer solution is expected to be a promising polymer material for application in energy storage devices, such as piezoelectric nanogenerators and capacitors.

## Figures and Tables

**Table 1:** Detailed experimental conditions for poly(vinylidenefluoride-co-trifluoroethylene) (P[VDF–TrFE]) thin film deposited via AP plasma deposition from the vaporized polymer precursor using a mixed polymer solution containing P[VDF–TrFE] polymer nano powder and dimethylformamide (DMF) solvent.

Precursor Polymer Solution	5% P[VDF–TrFE] Polymer Nano Powder + 95% DMF Solvent
Voltage (V_p_)	12.5 kV (fixed)
Frequency	26 kHz (fixed)
Ar gas flow for polymer vapor	450 sccm (fixed)
Ar gas flow	2500 sccm (fixed)
Bluff-body height	15 mm (fixed)
Length of the glass guide tube	Case I: 60 mm
	Case II: 80 mm
Deposition time	1 h (fixed)
Deposition temperature	Room temperature (fixed)
Post-heating temperature	140 °C, 160 °C, 180 °C (controllable)
Post-heating time	3 h (fixed)

**Table 2:** Detailed peak assignments of P[VDF–TrFE] solid nano powder and the deposited P[VDF–TrFE] thin films prepared via AP plasma deposition from the vaporized polymer precursor using a mixed polymer solution comprising P[VDF–TrFE] polymer nano powder and DMF liquid solvent in Figure 3.

Wavenumber (cm^–1^)		Peak Assignment
P[VDF–TrFE] Powder	P[VDF–TrFE] Thin Film	
850 cm^−1^		CF_2_ symmetric stretching (β-phase)
880 cm^−1^		CF_2_ in-plane rocking deformations (β-phase)
1288 cm^−1^	1288 cm^−1^	CF_2_ symmetric stretching (β-phase)
1400 cm^−1^	1400 cm^−1^	CH_2_ bond and C-C asymmetric stretching CH_2_ wagging vibration (β-phase)

**Table 3:** The obtained dielectric constant values of the post-heated P[VDF–TrFE] thin film presented in Figure 7.

Frequency	Capacitance (nF)	Dielectric Constant	Dielectric Loss
100 Hz	47	564	0.09
120 Hz	31	372	0.07
1 kHz	16	192	0.05
10 kHz	2.5	30	0.04

## Data Availability

Not applicable.
